# A combination of Bohr and Haldane effects provide a physiologic explanation for the increase in arterial oxygen saturation when a face mask is added to a high-flow nasal cannula in severely hypoxemic COVID-19 patients

**DOI:** 10.1186/s13054-021-03791-3

**Published:** 2021-11-16

**Authors:** Pablo Born, Ricardo Castro

**Affiliations:** Departamento de Medicina Intensiva, Unidad de Paciente Crítico – Hospital Clínico UC-CHRISTUS, Pontificia Universidad Católica de Chile, Diagonal Paraguay #362, Piso 6, Santiago, RM Chile

## Dear editor:

We read with great interest the article by Dogani et al. on the potential effect of adding an oxygen mask –without supplemental oxygen– to a high-flow nasal cannula on the improvement of oxygenation, in patients with acute hypoxemic respiratory failure due to COVID-19 [1]. We would like to share some comments considering: (a) the improbability that the oxygenation improvement could be related to a real rise in PaO_2_ by the use of a mask (although neither PaO_2_ nor pH data were provided), and (b) the lack of a straightforward physiological mechanism for this observation. We think that the explanation for the observed increase in the arterial oxygen saturation (SaO_2_) reported was due to a left shift on the hemoglobin dissociation curve, solely under the operation of Bohr and Haldane principles [2, 3].

**Fig. 1:**
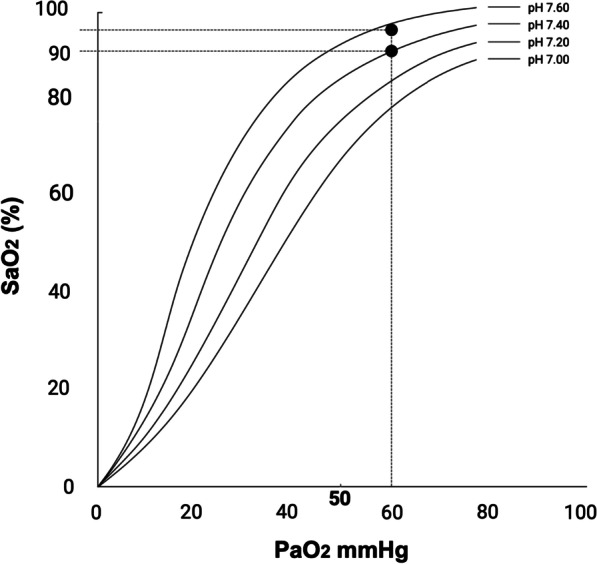
Hemoglobin dissociation curves according to acid–base status. Figure shows the arterial oxygen saturation (SaO_2_) variation (approximate values for the study by Dogani [1]) for a given PaO_2_ depending on arterial pH. *Note*: Figures drawn by Ricardo Castro

**Fig. 2:**
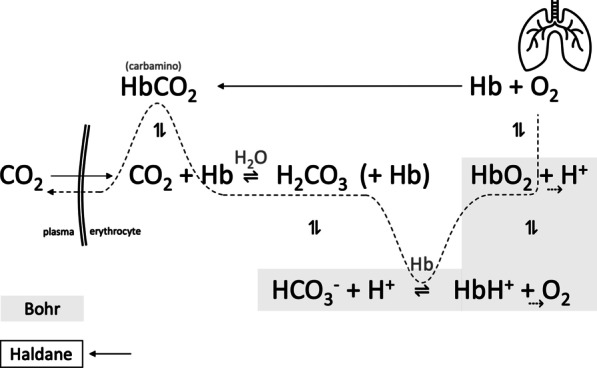
Haldane and Bohr effects

We propose that an improvement in SaO_2_ with a stable PaO_2_ came secondary to a rising in arterial pH according to the Bohr effect, or to a displacement of CO_2_ bound to hemoglobin (the carbamino compound) by an increase on blood oxygenation (Haldane) (Figs. 1 and 2), while keeping the arterial CO_2_ content relative stable. In fact, final PaCO_2_ remained unchanged in the eighteen patients of the study.

If we assume an increase in alveolar ventilation by a more efficient high-flow nasal cannula performance (concurring with the authors on a significant reduction of ambient air entrainment), plasma alkalinization came forth naturally: alongside with a ventilatory-driven PaCO_2_ decrease, protonated hemoglobin released H + which increased its affinity for O_2_ (the Bohr effect), increasing SaO_2_ consequently. On the CO_2_ metabolic side, this effect shifted the bicarbonate buffer equilibrium towards CO_2_ formation from carbonic acid, producing the release of this gas from erythrocytes that resulted in a slight rise in PCO_2_. Furthermore, as hemoglobin carried more oxygen, amino group-bound CO_2_ became displaced into its dissolved fraction (Haldane effect), all of which induced an increase in PaCO_2_ which would match the previous ventilatory PaCO_2_ decrease. As a final result, a slight or no major change in PaCO_2_ was observed.

Moreover, progressive hypoxemia correction may have produced an adaptive decrease on 2,3-diphosphoglycerate which left-shifted the hemoglobin dissociation curve further [4].

We acknowledge that we are presenting a theoretical model attempting to fill the gaps of an objectively documented observation, despite some lacking data. However, the combination of available data with classic physiologic principles provides another explanation for this interesting and clinically relevant phenomenon.

## Data Availability

Figures were drawn by the author/RC).
