# Supplementary material for: A combination of Bohr and Haldane effects provide a physiologic explanation for the increase in arterial oxygen saturation when a face mask is added to a high-flow nasal cannula in severely hypoxemic COVID-19 patients
Source: Crit Care. 2021 Nov 16;25:395. doi: 10.1186/s13054-021-03791-3 (PMC8593628; doi:10.1186/s13054-021-03791-3)
Supplement: Related texts

# Reply: Authors’ response letter

**Authors:** Besarta Dogani, Fredrik Månsson, Fredrik Resman, Hannes Hartman, Johan Tham, Gustav Torisson

Dear editor,

We thank Dr. Born and Dr. Castro for the input on our research letter describing an increased SaO_2_ when adding a mask to high-flow nasal cannula (HFNC) in patients with severe COVID-19 [1]. In a well-reasoned argument, Dr. Born and Dr. Castro propose that the increase in SaO_2_ may be due to a left shift in the hemoglobin dissociation curve, i.e. an increase in SaO_2_ with a stable PaO_2_ [5]. To expand the discussion, we would like to provide data regarding PaO_2_ and pH, from the arterial blood gases taken at baseline and after 30 min with mask.

In all participants, PaO_2_ increased after 30 min, with a mean difference of 3.2 KPa (95% CI 2.2–4.2), equivalent to ~ 24 mmHg (95% CI 16–32), see Fig. 3. This result is in line with the study by Montiel et al., who applied a surgical mask on top of HFNC [6]. In their study, PaO_2_ increased in all participants as well, with a mean difference of 20 mmHg (95% CI 13–26). Furthermore, in our study there was a slight decrease in the average pH after 30 min, from 7.480 to 7.471, with a mean difference of − 0.009 (95% CI − 0.018 to − 0.001). Therefore, we consider it unlikely that a left shift in the hemoglobin dissociation curve would fully explain the observed phenomenon.

**Fig. 3:** Partial pressure of oxygen (PaO_2_) in KPa from arterial blood gases taken at baseline and after 30 min with mask. error bars = 95% confidence interval of the mean. *** = *p* value < .001 from paired *t*-test

We hypothesised that the mask could minimise entrainment of room air, especially when mouth-breathing, although we have no data to support this hypothesis. To the best of our knowledge, no studies have measured fiO2 in COVID-19 patients with HFNC. Studies in healthy volunteers suggest that fiO2 may decrease upon mouth-breathing or when exercise was used to simulate respiratory distress [7]. Whether this applies in COVID-19 is uncertain.

It was beyond the scope of our research letter to study the underlying mechanism, and several concurrent processes may contribute. Therefore, we are sincerely grateful for all input in this matter, including that of dr Swenson [8]. Hopefully, further research in oxygen delivery in severe COVID-19 will elucidate this matter.
